# Critical view of anaphylaxis epidemiology: open questions and new perspectives

**DOI:** 10.1186/s13223-018-0234-0

**Published:** 2018-04-04

**Authors:** Luciana Kase Tanno, Ana Luiza Bierrenbach, F. Estelle R. Simons, Victoria Cardona, Bernard Yu-Hor Thong, Nicolas Molinari, Moises A. Calderon, Margitta Worm, Yoon-Seok Chang, Nikolaos G. Papadopoulos, Thomas Casale, Pascal Demoly, on behalf the Joint Allergy Academies

**Affiliations:** 1 Hospital Sírio Libanês, São Paulo, Brazil; 2 University Hospital of Montpellier, Montpellier, France; 3 Sorbonne Université, INSERM, IPLESP, 75013 Paris, France; 4 Sanas Epidemiology and Research, São Paulo, Brazil; 5 Teaching Research Institute (IEP), Hospital Sírio Libanês, São Paulo, Brazil; 6 Section of Allergy & Clinical Immunology, Department of Pediatrics & Child Health, University of Manitoba, Winnipeg, Canada; 7 Allergy Section, Department of Internal Medicine, Hospital Universitari Vall d’Hebron, Barcelona, Spain; 8 Department of Rheumatology, Allergy and Immunology, Tan Tock Seng Hospital, Singapore, Singapore; 9 IMAG, UMR 5149, DIM CHRU de Montpellier, Montpellier, France; 10 Section of Allergy and Clinical Immunology, Imperial College London, National Heart and Lung Institute, Royal Brompton Hospital, London, UK; 11 Allergie-Centrum-Charité, Klinik für Dermatologie, Venerologie und Allergologie, Universitätsmedizin Berlin, Campus Charité Mitte, Berlin, Germany; 12 Division of Allergy and Clinical Immunology, Department of Internal Medicine, Seoul National University Bundang Hospital, Seoul National University College of Medicine, Seongnam, Gyeonggi-do South Korea; 13 Centre for Paediatrics and Child Health Institute of Human Development, University of Manchester, Manchester, UK; 14 Department of Allergy, 2nd Pediatric Clinic, University of Athens, Athens, Greece; 15 American Academy of Allergy Asthma and Immunology, and Morsani College of Medicine, University of South Florida, Tampa, FL USA; 16 Division of Allergy, Department of Pulmonology, Hôpital Arnaud de Villeneuve, University Hospital of Montpellier, 371, av. du Doyen Gaston Giraud, 34295 Montpellier Cedex 5, France

**Keywords:** Anaphylaxis, Classification, Epidemiology, International Classification of Diseases, Rare diseases, World Health Organization

## Abstract

In contrast to the majority of allergic or hypersensitivity conditions, worldwide anaphylaxis epidemiological data remain sparse with low accuracy, which hampers comparable morbidity statistics. Data can differ widely depending on a number of variables. In the current document we reviewed the forms on which anaphylaxis has been defined and classified; and how it can affect epidemiological data. With regards to the methods used to capture morbidity statistics, we observed the impact of the anaphylaxis coding utilizing the World Health Organization’s International Classification of Diseases. As an outcome and depending on the anaphylaxis definition, we extracted the cumulative incidence, which may not reflect the real number of new cases. The new ICD-11 anaphylaxis subsection developments and critical view of morbidity statistics data are discussed in order to reach new perspectives on anaphylaxis epidemiology.

## Anaphylaxis epidemiology: open introductory questions

Anaphylaxis has been defined for clinical use by healthcare professionals as a serious, generalized, allergic or hypersensitivity reaction that can be life-threatening and even fatal [1–3]. In contrast to the majority of allergic or hypersensitivity conditions such as asthma or rhinitis, accurate worldwide anaphylaxis epidemiological data remain lacking for harmonization. Data can differ widely depending on a number of variables. For instance, European data have indicated incidence rates for all-cause anaphylaxis ranging from 1.5 to 7.9 per 100,000 person/year, with an estimate that 0.3% (95% CI 0.1–0.5) of the population will experience anaphylaxis at some point during their lifetime [4]. As well, it is estimated that 1 in every 3000 inpatients in US hospitals suffer from an anaphylactic reaction [5].

**Fig. 1:**
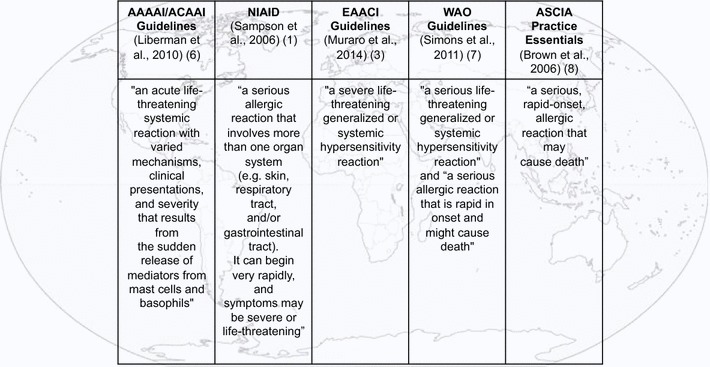
Similarities and differences in anaphylaxis definitions worldwide [1, 3, 6−8]

Although available data, specifically those collected during the past decade, show an increased frequency of anaphylaxis, there are still challenges in interpreting these informations [6, 7] and its global applicability. The most widely discussed issues in the epidemiology of anaphylaxis filed over the last 10 years are: (I) regional variations in concepts and definitions (Fig. 1) [1–3, 8–10], (II) whether prevalence or incidence is the best measure of the frequency of anaphylaxis in the general population, (III) whether the frequency of anaphylaxis is higher than previously thought, and (IV) whether the increasing incidence published is real or reflects different methodologies and definitions used.

**Fig. 2:**
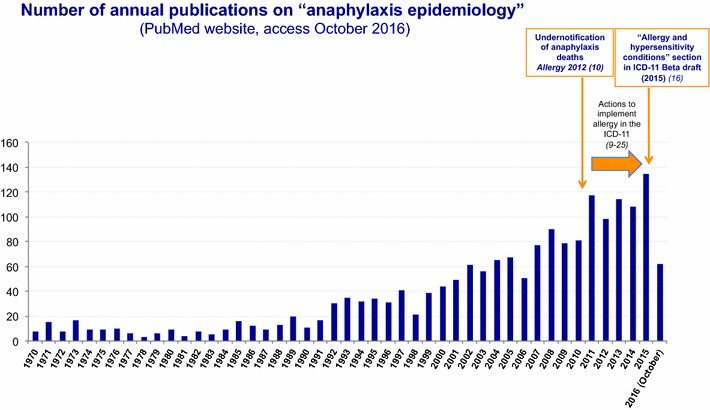
Annual number of publications retrieved from Pubmed Central^®^ using the search term “anaphylaxis epidemiology”, (accessed November 7, 2016)

Over the last several years, an increasing number of clinical databases have been developed to capture reliable anaphylaxis epidemiological data at both national and regional levels (Fig. 2). However, a substantial proportion of the current data on the epidemiology of anaphylaxis has come from registries with limited scope and population source. Different methods have been applied in an attempt to reach reliable epidemiological data, but most of the studies have focused on specific triggers or at-risk populations. Lack of harmonized strategies to record anaphylaxis cases hampers collection of comparable epidemiological data. In general, registries are representative sources to reach epidemiological data, and are applied only if the reporting of the conditions is mandatory and the data are validated.

On the other hand, broad population-based studies allow descriptive and analytic epidemiological analysis covering the general population and identify all or a known fraction of the cases in a particular community. Generally speaking, population-based studies have adopted the World Health Organization (WHO) International Classification of Diseases (ICD) as the main tool to capture the proposed outcomes. These studies have been especially useful in detailing time-related trends, capturing clinical practice and principal discharge diagnosis coding statistics, and in providing broader morbidity and mortality statistics (MMS). However, anaphylaxis has never been well classified under the ICD context, either for morbidity [11] (Table 1) or for mortality data [12]. This is exemplified by the under-notification of anaphylaxis deaths using the Brazilian national mortality database [12]. An important reason for this is the difficulty of coding anaphylaxis fatalities under the WHO ICD system. In most countries, mortality statistics are routinely compiled according to regulations and recommendations adopted by the World Health Assembly (WHA). Causes of deaths are classified and grouped according to the ICD edition in use at the time and the information on death certificates is collected using the international form recommended by the WHO. However, a limited number of ICD-10 codes are considered to be valid for representing underlying causes of death on the current death certificates, and with regard to anaphylaxis as such, there are simply no valid codes [12].

**Table 1:** Anaphylaxis classification and coding in ICD-10 and in ICD-11

Anaphylaxis ICD-10 codes (2016 version)		Anaphylaxis ICD-11 codes (November 2016 version)
Chapter XIX injury, poisoning and certain other consequences of external causes (S00–T98)		Chapter 04 disorders of the immune system
Other and unspecified effects of external causes (T66–T78)		Section allergic and hypersensitivity conditions
T78 Adverse effects, not elsewhere classified Note: This category is to be used as the primary code to identify the effects, not elsewhere classifiable, of unknown, undetermined or ill-defined causes. For multiple coding purposes this category may be used as an additional code to identify the effects of conditions classified elsewhere		Subsection anaphylaxis
T78.0 Anaphylactic shock due to adverse food reaction		4B50 Anaphylaxis due to allergic reaction to food
T78.1 Other adverse food reactions, not elsewhere classified		4B51 Drug-induced anaphylaxis
T78.2 Anaphylactic shock, unspecified		4B52 Anaphylaxis due to insect venom
Allergic shock	NOS	4B53 Anaphylaxis provoked by physical factors
Anaphylactic reaction		4B53.1 Exercise-induced anaphylaxis
Anaphylaxis		4B53.2 Cold-induced anaphylaxis
T78.3 Angioneurotic oedema		4B53.Y Anaphylaxis provoked by other specified physical factors
Giant urticaria		4B53.Z Anaphylaxis provoked by unspecified physical factors
Quincke oedema		4B54 Anaphylaxis due to inhaled allergens
T78.4 Allergy, unspecified		4B55 Anaphylaxis due to contact with allergens
		4B56 Anaphylaxis secondary to mast cell disorder
Allergic reaction NOS		
Hypersensitivity NOS		4B5Y Other specified anaphylaxis
Idiosyncracy NOS		
T78.8 Other adverse effects, not elsewhere classified		4B5Z Anaphylaxis, unspecified
T78.9 Adverse effect, unspecified		

Taking the opportunity presented by the ongoing ICD-11 revision, the under-notification of death data [12] triggered a cascade of strategic international actions supported by the Joint Allergy Academies and the ICD WHO governance [11–23] to update the classifications of allergic conditions for the new ICD edition. These efforts have resulted in the construction of the new “Allergic and hypersensitivity conditions” section built under the “Disorders of the Immune system” chapter [17, 24].

In order to better delineate the proposed changes and follow the ICD-11 revision agenda, we reviewed the forms on which anaphylaxis has been defined and classified, and the published anaphylaxis epidemiological data, particularly with regards to the methods used to capture morbidity statistics.

## Anaphylaxis: reaching answers based on definition and classification

### Anaphylaxis definition impacts in epidemiological data

**Fig. 3:**
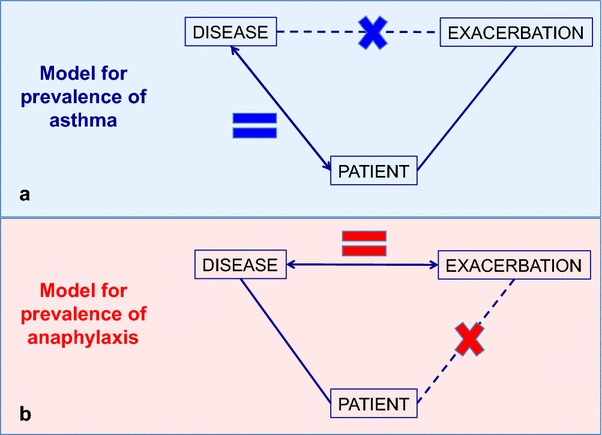
General model to explain how definitions of allergic conditions can affect prevalence data (**a** asthma as a model, **b** anaphylaxis as a model)

All anaphylaxis guidelines [1–3, 8–10] have consistently defined anaphylaxis as a severe life-threatening generalized or systemic hypersensitivity reaction. Being described as a reaction implies a risk of overestimating the prevalence of anaphylaxis. For some conditions, such as asthma, the number of patients with the condition is different from the number of (asthma) exacerbations (Fig. 3a). However, using the definition of anaphylaxis, the number of exacerbations (reactions) can be wrongly taken as the number of cases (disease) (Fig. 3b), resulting in an overestimated lifetime cumulative incidence.

Incidence is the measure of the frequency of a new case (or condition) in a population at risk within a period of time. Therefore, given that anaphylaxis is an acute condition with long asymptomatic periods during which the risk of relapse can decrease over time in some patients or when culprit factors such as allergens are avoided or counteracted (e.g. stinging insect venom immunotherapy), knowing the number of new episodes (incidence) over a specific period can not be an adequate measure of frequency. Cumulative incidence, also known as incidence proportion may be not an adequate measure for frequency of anaphylaxis, since the reaction is no longer active once the episode is resolved. For this reason, more detailed data on recurrent episodes are required to overcome this challenge in anaphylaxis epidemiology studies.

### Updated anaphylaxis classification and coding in the ICD

The ICD is the broadest classification and coding system used to monitor the incidence and prevalence of diseases and other health problems, reflecting the general health situation in countries and populations [25].

Taking the example of the ICD-10 (2016 version) [26], anaphylaxis has been classified under the “XIX Injury, poisoning and certain other consequences of external causes” chapter, specifically the “T78 Adverse effects, not elsewhere classified” section. Under this section, different hypersensitivity conditions are classified at the same level, such as “Anaphylactic shock due to adverse food reaction”, “Angioneurotic oedema” and “Allergy, unspecified” (Table 1), reflecting a misunderstanding of concepts used by the health care professionals on a daily basis.

The construction of the new ICD-11 section addressing to allergic and hypersensitivity conditions now allows anaphylaxis to be properly classified and attaining greater visibility within ICD (Table 1). Currently, this subsection contains 7 main anaphylaxis headings to be combined with severity and causality classification/specifications. The building process of this framework resulted from combined efforts and constant discussions with the groups of experts and the ICD WHO governance.

The construction of the new subsection addressed to anaphylaxis means that it will now be recognized as a clinical condition requiring specific documentation and management.

### Lessons from population-based anaphylaxis morbidity epidemiology publications

**Fig. 4:**
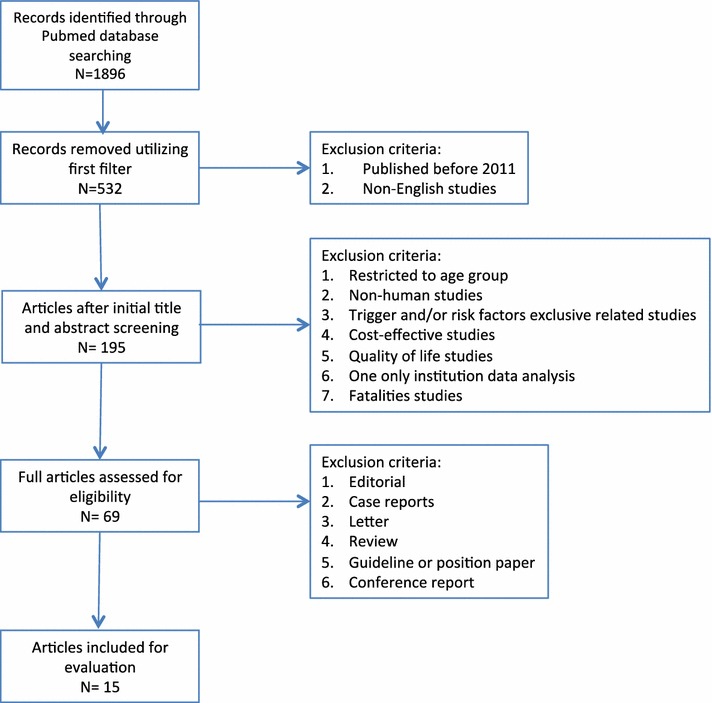
Anaphylaxis epidemiology publications eligibility selection process

From 19th to 26th October 2016, 1896 manuscripts were selected using PubMed Mesh terms “anaphylaxis epidemiology”, “epidemiology of anaphylaxis” or “anaphylaxis epidemiology population-based studies”. After removing those published before 2011 and those not published in English, there were 532 papers published in the last 5 years. We did not include case reports, studies in animal models, quality of life studies, fatality studies, guidelines or reviews (Fig. 4). All publications were independently evaluated by two co-authors and disagreements related to the inclusion into the analysis were resolved through open discussion and consensus.

We analyzed methodological aspects, main outcomes and databases used in the remaining 15 publications selected as eligible according to the selection criteria (Fig. 4) from different countries. The methods used and the definitions taken varied among the publications; however, 67% focused on rates of hospitalization or emergency department admissions. National databases were used in 67% of the studies. Overall, 40% were large population-based studies and 100% of these documents used the ICD definition as the starting point of the analysis (Table 2). Based on ICD registries, regardless of the ICD version used, 71% of all the studies had to utilize secondary data in order to capture the anaphylaxis data, meaning that the data have been affected by the misclassification of anaphylaxis in the previous versions of the ICD.

**Table 2:** Anaphylaxis morbidity epidemiology publications

References	Study location	Study design and anaphylaxis definition chosen	Epidemiological variables suited and outcomes	Number of subjects	Study period	Study anaphylaxis morbidity database
Beyer et al. [31]	Germany	Self-reported cases from the general population based on survey questionnaire	Incidence of severe anaphylaxis: 4.5 per 100,000	333	2008–2010	Local database
Buka et al. [32]	United Kingdom	Retrospective observational study based on search terms considering the WAO anaphylaxis definition [7]	Incidence of anaphylaxis: 12.0 per 100,000 person-years	426 patient episodes	12 months (2012)	National Health Service (NHS) database
Campbell et al. [33]	United States of America	Historic-prospective observational cohort study with analysis of medical records based on the NIAID/FAAN diagnostic criteria for anaphylaxis [1]	Prevalence of anaphylaxis: 582 Patients needing more than 1 dose of epinephrine: 8%	582	2010–2013	National SAS-Display Manager System database
Cetinkaya et al. [34]	Turkey	Search for ICD-10 anaphylaxis and secondary codes as primary cause of hospitalization	Incidence admissions for anaphylaxis: 11.05 per 100,000 person-years	114 patients	12 months (2010–2011)	National database: The Health Directorate Network of Istanbul
Gibbison et al. [35]	United Kingdom	Retrospective analysis of all physician-diagnosed cases in national audit data from critical care units considering NIAID/FAAN anaphylaxis definition [1] as first or secondary cause of admission	Paediatric admissions with anaphylaxis: 6–23/year Adult admissions with anaphylaxis: 183–331/year	1350 admissions for anaphylaxis	2005–2009	National audits of critical care units in the UK: the Intensive Care National Audit and Research Centre’s (ICNARC) Case Mix Programme (CMP), Scottish Intensive Care Society Audit Group (SICSAG) and Paediatric Intensive Care Audit Network (PICANet)
Grunau et al. [36]	Canada	Retrospective cohort study with dichotomized algorithm for “anaphylaxis” considering the WAO definition for anaphylaxis [7]	Emergency department visits for anaphylaxis: 23.2 per 100,000 person-years	496	2007–2012	Local electronic medical records database
Harduar-Morano et al. [37]	United States of America	Population-based epidemiologic study using national database and selected ICD codes	Incidence of anaphylaxis: 7.5 per 100,000 person-years	2751	2005–2006	Agency for Health Care Administration
Jeppesen et al. [38]	Denmark	Population-based cohort study using national database and selected ICD codes	Average hospitalization rate for first-time anaphylactic shock was 64.6 per 1,000,000 person-years	6707	1995–2012	Danish National Patient Registry and the Danish Civil Registration System
Kimchi et al. [39]	Canada	Software program applied to prospectively record characters of anaphylaxis cases taking the WAO definition of anaphylaxis [7]	% Anaphylaxis among all calls: 0.31 % Anaphylaxis among calls requiring transport: 0.44	104	12 months (2013–2014)	Cross Canada Anaphylaxis Registry database
Lee et al. [40]	United States of America	Population-based incidence study selected by ICD codes	Incidence rate of anaphylaxis was 42 per 100,000 person-years	2386	2001–2010	Rochester Epidemiology Project linked to the list of all Olmsted County residents
Mullins et al. [41]	Australia	Population-based epidemiologic study using national database and selected ICD codes	Increase in overall anaphylaxis admission rates: 1.5-fold/7 years	3.6–17.7 cases/10^5^ population	2005–2006 and 2011–2012	Australian National Hospital morbidity database
Tejedor Alonso et al. [42]	Spain	Population-based epidemiologic study using national database and selected ICD codes	Anaphylaxis admissions in the general population: 1.35–2.38 per 100,000 person-years	11,336 records	1998–2011	Spanish information system for hospital data
Wood et al. [43]	United States of America	Self-reported cases from the general population based on survey questionnaire considering the main anaphylaxis definitions [1, 3, 6, 7]	Prevalence of anaphylaxis in the general population: 1.6%	344	5 months (2011)	Local database
Worm et al. [44]	European countries (Austria, Bulgaria, France, Germany, Greece, Ireland, Italy, Poland, Spain and Switzerland)	Data collection from specialized European countries by online questionnaire considering the EAACI-WAO revised nomenclature [45]	Number of anaphylaxis cases: 3333 Recurrence: 34.2%	3333	2011–2014	European anaphylaxis registry
Turner et al. [45]	United Kingdom	Population-based epidemiologic study using hospital admission data and selected ICD codes	Number of hospital admissions for anaphylaxis: 1.0–7.0 cases per 100,000 population per annum	Data limited to the number of admissions	1992–2012	Hospital episodes statistics database

Most of the studies (60%) did not address the possibility of recurrence of episodes, and, therefore, of cumulative incidence. This highlights the need to distinguish the number of patients with anaphylaxis per year from the number of episodes per year. Even with the alternative strategy of reaching the mean number of anaphylaxis cases within a started time, data would be influenced by the methodology and definitions applied. In other words, most of epidemiological studies considering incidence as the main variable may be overestimating the number of anaphylactic patients. True frequency of anaphylaxis is also possibly underestimated due to under-recognition by patients and caregivers and under-diagnosis by health care professionals (e.g.: difficulty on diagnosing anaphylaxis in the absence of hypotension or shock).

## Reaching new perspectives for anaphylaxis epidemiology

In this manuscript, we provide arguments for the need of reviewing the current definitions in use for anaphylaxis. The definitions are able to directly impact in the epidemiology of anaphylaxis as a disease. Incorporating refined strategies to achieve accuracy and comparable MMS can support public health changes to reach better patients’ care and prevention worldwide.

Due to recent achievements at the international level on enhancing terminology, classification, definitions and coding of allergic and hypersensitivity conditions through the ongoing WHO ICD revision process, anaphylaxis is now considered a condition. Importantly the new classification system for anaphylaxis will surely enable the collection of more accurate epidemiological data to support quality management of patients with allergies, better health care planning and decision-making and public health measures to reduce the morbidity and mortality attributable to anaphylaxis.

Based on current developments, reviewing the definition of anaphylaxis and epidemiology strategies by clarifying differences between anaphylaxis episodes (reactions) from anaphylaxis as a condition itself will lead to more precision on MMS. Cumulative incidence is an inadequate measure here, since the reaction is no longer active once the episode is resolved and one patient can present different episodes of anaphylactic reactions in his/her life. Most of the publications have so far considered the episodes (illness), regardless to the subject suffering of this condition (patient). A possible strategy in order to avoid cumulative incidence of anaphylaxis would be reaching recurrence of anaphylactic episodes. Consistent scientific, economical and political changes may follow this move, which will likely be reflected in better management of patients with anaphylaxis worldwide. For instance, precise and broader anaphylaxis MMS will support the global availability of auto-injectable adrenaline, currently available in less than 35% of all countries [23]. Specific focus in the patients’ care, and not just in the episodes, would also support primary and secondary prevention actions.

As demonstrated, anaphylaxis regional epidemiological data differ considerably according to many variables and it is still unclear whether the increasing incidence published is real or the results reflect different methods used to define and characterize anaphylaxis. However, based on current statistics [4, 5], severe anaphylaxis fits well the definition of a rare disease. Conceptually, rare diseases can be defined as life-threatening or chronic debilitating disorders, which are of low prevalence and typically require a multi-disciplinary approach to address prevention and treatment. The Orphanet, lead by the French National Institution of Health and Medical Research (INSERM) and the French Ministry of Health, is responsible for developing an inventory of rare diseases and a classification system which could serve as a template to update International terminologies. When the WHO launched the revision process of the ICD, a rare diseases Topic Advisory Group was established. So far 5400 rare diseases listed in the Orphanet database have an endorsed representation in the foundation layer of ICD-11 [27, 28], but severe anaphylaxis is not yet included on the list. In addition to all the benefits expected by the actions to update terminology, definitions and classification of allergic and hypersensitivity conditions through the ICD-11 revision, we strongly believe that anaphylaxis is a public health priority, therefore in order to strengthen awareness and quality clinical management of patients it should therefore be formally added to the list of rare diseases. Including severe anaphylaxis into the list of rare diseases may allow, in first instance, the allocation of resources to better understand the national and global epidemiology of anaphylaxis as a disease; monitor the patterns of this disorder to follow hospitalizations, mortality, avoidable deaths and costs. Having more precise epidemiological data may support the global availability of adrenaline auto-injectors worldwide at affordable price addressed to the patients’ care through argumentation with national bodies and stakeholders. For instance, the French Ministry of Health, in coordination with the State Secretariat for Higher Education and Research, implements a proactive policy based on the mobilization of health/research professionals and patients’ associations in order to improve quality diagnosis, management and prevention of the 3 million patients affected by rare diseases in France. This health intervention/policy model can be took as an example in different countries in order to implement essential actions according to individual national needs, such as the availability of adrenaline auto-injectors in low incoming countries [23].

Anaphylaxis epidemiological publications are also hampered by the inclusion of all severity degrees of anaphylaxis. Mild reactions in which manifestations are generally limited to one organ or system, such as the skin, usually do not incur any risk of death. The inclusion of these cases in the epidemiological studies provides mistaken perception of high and increasing incidence of severe anaphylaxis. Our focus would be addressing severe reactions in which the risk of mortality is strong and requires additional prevention measures and a coordinated management such as the one provided by the rare disease network in our country. To date, there is no available data regarding severe cases of anaphylaxis in Europe. French data suggests that less than 30,000 people are affected by severe anaphylaxis and, 9.2 per 100,000 person-years based on the University Hospital of Montpellier data [29]. Australian data demonstrated the increasing number of patients at risk of anaphylaxis, from 0.98% in 2009 reaching 1.38% in 2014 in school aged children. In contrast, the number of adrenaline auto-injectors activated (severe cases) per year per 1000 students at risk of anaphylaxis was 6 and 8 in 2010 and 2014 respectively [30]. If taken as isolated data of patients at risk, it can drive readers to think that anaphylaxis is increasing in this country. However, the administration of the treatment as objective data indicates that severe anaphylaxis can be considered as rare disease.

The ICD-11 intends will be presented to the WHA in 2018. A known issue regarding accurate epidemiological data and miscoding is the lack of training regarding how disorders should be classified and coded in administrative and institutional databases. For this reason, the core ALLERGY in ICD-11 operational team (LKT, PD) in collaboration with the WHO and with the support of our international academies network, intends to implement education tools to support the allergy community in the transition process, preparing health professionals and stakeholders for the new logic of the ICD-11, when it is launched. Educational efforts will also help to decrease the under-recognition of anaphylaxis by patients, caregivers, and health professionals, health authorities and governments and have been the main aim of allergy academies by promoting education programs and publications in the field.

The construction of the new section dealing with anaphylaxis means that the latter will now be recognized as a clinical condition requiring specific documentation and management. Besides increasing the accuracy and sensitivity of clinical diagnosis data, unifying the allergic and hypersensitivity conditions into a single section of the ICD, endorsed by the WHO ICD governance bodies can be considered a strong epidemiological, economical and political move that advocates to optimal diagnosis and management of allergic patients worldwide. By allowing all the relevant diagnostic terms for anaphylaxis to be included into the ICD-11 framework, WHO has recognized their importance not only to clinicians but also to epidemiologists, statisticians, health care planners and other stakeholders. In the current manuscript we raise awareness of the outcomes of the ongoing ICD revision process as an instrument that has been developed to provide more precise anaphylaxis MMS to ensure comparability in monitoring, decision-making and achieving quality clinical practice. Meanwhile we propose strategies to improve anaphylactic patients’ care through reviewing definitions and epidemiological data. This document and critical view intend to support national and international health interventions and health policy changes.

## Abbreviations

AAAAI: American Academy of Allergy Asthma and Immunology
ACAAI: American College of Allergy Asthma and Immunology
ASCIA: Australian Society of Clinical Immunology and Allergy
EAACI: European Academy of Allergy and Clinical Immunology
ICD: International Classification of Diseases
MMS: morbidity and mortality statistics
NIAID/FAAN: National Institute of Allergy and Infectious Disease/Food Allergy and Anaphylaxis Network
WAO: World Allergy Organization
WHO: World Health Organization
